# THE INFLUENCE OF SPIRITUALITY AND RELIGION ON NURSING HOME SOCIAL WORKERS PROFESSIONAL IDENTITY AND RESILIENCE

**DOI:** 10.1093/geroni/igad104.3649

**Published:** 2023-12-21

**Authors:** Brianna Garrison, Dennis Myers

**Affiliations:** Southern Connecticut State University, New Haven, Connecticut, United States; Baylor University, Waco, Texas, United States

## Abstract

The complexities of social workers’ behavioral integration of personal spirituality within nursing homes, where a highly regulated, stressful environment marginalizes professional identity and challenges retention, remains largely unexplored and untested. The paucity of data limits the profession’s commitment to resourcing current and future practitioners with evidence-informed integration of spirituality with professional identity, ethical practice, and continuation in the role. This qualitative study addresses this gap by interviewing and analyzing narratives authored by experienced NHSWs who daily navigate the spirituality-at-work opportunity and challenge. Researchers collected a purposive sample of licensed NHSWs working within these settings and consenting to participate. All respondents held a social work license at the baccalaureate or master’s level (LBSW, LMSW, or LCSW) and were employed as a social worker full time for an average of 3.2 years in their current nursing facility and their overall experience in nursing facility social work averaged 8.8 years, with a range of 2 to 30 years’ experience. Twenty NHSWs were interviewed and 80% (n=16) associated their role, at some level, with a higher power (God, Christ, Holy Spirit, unnamed) and religious beliefs and practices. Fifteen percent (n=3) stated faith and religious ideology had no impact or was irrelevant for practice, and one (5%) did not respond. It became clear that religion, spirituality, and faith practices matter for a majority of the respondents in how they view and enact their practice.

